# Building trust and inclusion with under-served groups: a public involvement project employing a knowledge mobilisation approach

**DOI:** 10.1186/s40900-024-00647-2

**Published:** 2024-11-11

**Authors:** Anna M. Anderson, Lucy Brading, Laura Swaithes, Nicola Evans, Sophia E. Fedorowicz, Darren Murinas, Elizabeth Atkinson, Alice Moult, Tatum Yip, Parveen Ayub, Krysia Dziedzic, Philip G. Conaghan, Gretl A. McHugh, Amy Rebane, Sarah R. Kingsbury

**Affiliations:** 1 Leeds Institute of Health Sciences, University of Leeds, Leeds, UK; 2 Leeds Institute of Rheumatic and Musculoskeletal Medicine, University of Leeds, Leeds, UK; 3 Impact Accelerator Unit, Keele University, Keele, UK; 4 Expert Citizens CIC, Stoke-On-Trent, UK; 5 NIHR Leeds Biomedical Research Centre, Leeds, UK; 6 Healthwatch Leeds, Leeds, UK; 7 School of Healthcare, University of Leeds, Leeds, UK

**Keywords:** Public involvement, Public partnership, Community engagement, Inclusion, Trust, Under-served groups, Knowledge mobilisation, Community conversations, Co-production

## Abstract

**Background:**

Certain groups are commonly under-served by health research due to exclusionary models of research design/delivery. Working in partnership with under-served groups is key to improving inclusion. This project aimed to explore the use of a knowledge mobilisation approach to start building partnerships with under-served groups based on trust and mutual understanding.

**Methods:**

This co-produced public involvement project employed a knowledge mobilisation approach. The project team involved public contributors from four community organisations and staff from two Universities. A series of ‘community conversations’ were co-produced. These involved open discussions with local people in community settings. The conversations provide an informal space to engage in a multi-directional dialogue about health research and incorporated approaches such as prompt questions, live illustrations, and themed boards. The findings were reviewed collectively. Dissemination/feedback activities and lessons learned for future engagement with community organisations and under-served groups were also co-produced.

**Results:**

Over 100 people attended the community conversations. Attendees varied widely in their sociodemographic characteristics (e.g., socioeconomic status and ethnicity) and brought diverse lived experiences (e.g., experiences of homelessness and disability). A strong appetite for change and desire to mobilise public knowledge were evident. Attendees reported wide-ranging barriers to inclusion in health research and suggested ways to address them. Three inter-related take-home messages were identified: ensure relevance, appreciation, and trust; prioritise language and accessibility needs; and maximise flexibility in all research-related activities. Feedback about the community conversations and dissemination activities was largely positive, with all parties planning to continue the partnership building. The lessons learned provide practical suggestions for promoting inclusion in research and highlight the importance of addressing research teams’ training/support needs.

**Conclusions:**

Knowledge mobilisation was a valuable approach for facilitating multi-directional dialogues and relationship building between local communities and university teams. This approach enabled co-creation of new knowledge related to inclusion and partnership working in health research. The project has provided a firm foundation to build upon. However, creating sustainable, inclusive public partnerships is likely to require systemic changes, such as weighting of fundings schemes to projects that prioritise inclusion of under-served groups throughout the research cycle.

**Supplementary Information:**

The online version contains supplementary material available at 10.1186/s40900-024-00647-2.

## Abstract (plain language summary)

Some groups of people often face barriers to being involved in health research. Researchers need to get better at working with these under-served groups so that the groups’ voices contribute to research, and the groups benefit from research.

This project aimed to explore the use of a knowledge mobilisation approach to start building partnerships with under-served groups based on trust and mutual understanding. Knowledge mobilisation involves research teams and people beyond academia sharing what they know with each other. This helps to create new knowledge with practical benefits in the real world.

The project team involved people from four community organisations and two universities. Informal conversations with over 100 local people were held in community settings. Prompt questions and other activities were used to encourage people to share their views of health research. The conversations suggested research teams need to:

1. Work with local communities to design research that is relevant to them, show their input is valued, and build trust.
2. Meet people’s language and disability-related needs.
3. Make all research activities as flexible as possible.

This project’s lessons learned provide practical examples of how to make research more inclusive and highlight the importance of addressing research teams’ training/support needs.

Overall, knowledge mobilisation was a helpful approach for building relationships with under-served groups and working together to create new knowledge. All the groups involved plan to carry on working together. Keeping the partnerships going long term may need wider changes, for example in how research is funded.

**Supplementary Information:**

The online version contains supplementary material available at 10.1186/s40900-024-00647-2.

## Background

Equality, diversity and inclusion (EDI) has become a key strategic priority for health services and funding bodies. For example, in the United Kingdom (UK), the National Institute for Health and Care Research (NIHR) recently launched an EDI strategy [1] and Race Equality Framework [2]. These documents underpin the NIHR’s commitment to improving inclusion of under-served groups in health and social care research. The NIHR advocates use of the term ‘under-served’ based on the Innovations in Clinical Trial Design and Delivery for the Under-served (INCLUDE) project [3, 4]. This suggested the term ‘under-served’ helps to emphasise that certain groups of people are often under-represented in research due to exclusionary models of research design and delivery, rather than any fault of the groups themselves.

The INCLUDE project highlighted that the definition of under-served groups is highly context-specific, varying with factors such as a study’s target population and research question [3, 4]. Key characteristics of under-served groups include relatively low enrolment rates in studies; experiencing a high health burden but having few research opportunities; and not being offered suitable interventions during studies, despite being likely to respond to/engage in the interventions differently to other groups [3, 4]. Groups with certain demographic, socioeconomic, and health-related characteristics are commonly under-served by health research. These include people from Black African, Asian and Caribbean heritage communities; socially marginalised groups (e.g., people experiencing homelessness); and disabled people [2, 3].

Improving inclusion of under-served groups in health research is vital to ensure evidence-based care is acceptable, safe, and effective for everyone. Intentionally including under-served groups in health research is also key to understanding and reducing health inequities [5, 6]. Achieving this requires multifaceted strategies spanning areas such as research funding, the research workforce, and public partnerships [1, 7]. The NIHR considers public partnerships as an overarching term for any ways in which public contributors (e.g., patients, carers, and people from community organisations) work with researchers and health and care professionals to create and use research [7]. This encompasses activities in three inter-related areas: involvement (where research activities are carried out ‘with’ or ‘by’ public contributors), participation (where people take part in research), and dissemination (where information/knowledge about research is shared) [1, 7].

Although the importance of building inclusive partnerships with under-served groups is recognised, researchers do not always know the best ways to achieve this [8]. Furthermore, it takes substantial time and resources to build trusting, reciprocal relationships in a sustainable way [8, 9]. If people involved in health research do not feel listened to, they may become further disillusioned, perpetuating the belief that their voices are only needed to tick a box, rather than bring about meaningful changes [10, 11]. A growing body of literature provides suggestions for addressing these issues. For example, the NIHR recently published a guide to being inclusive in public involvement in health research [9, 12]. This was based on the NIHR Reaching Out programme, which involved four projects focused on developing relationships between research organisations/teams and local under-served communities [9].

The NIHR inclusive public involvement guide provides 12 prompts for researchers, along with learning points and practical examples [9, 12]. The first prompt, ‘Check your power’, is key due to the power imbalances between research teams and members of the public, with proactive steps being needed to start addressing power imbalances [9, 12]. Prompt 7 recommends that researchers collaborate with local community organisations, which typically have extensive experience of working with under-served groups and well-established, trusting relationships. The NIHR inclusive public involvement guide was developed based on interviews with the Reaching Out project leads, so may not directly reflect the views of community organisations or under-served groups [9]. In contrast, the CHecklist for Inclusive COmmunity involvement in health research (CHICO) guidance was co-produced by two researchers and members of three community organisations [13]. The CHICO guidance provides a range of recommendations spanning building relationships, reciprocal relationships, and practicalities. While the recommendations are likely to be helpful in various contexts, a limitation of the CHICO guidance is that all the organisations involved in developing it were Bristol-based organisations for people from minority ethnic groups. Correspondingly, the CHICO guidance authors describe their work as a 'launch pad for others to add to’ [13].

Both the NIHR inclusive public involvement guide [9, 12] and CHICO guidance [13] emphasise the importance of investing in relationship building with community organisations and individuals. Relationship building is also a key feature of knowledge mobilisation – a process in which research teams and people beyond academia share what they know with each other to co-create new knowledge that can make a practical difference in the real world [14–16]. Importantly, knowledge mobilisation recognises the value of various types of knowledge, including the experiential and embodied knowledge that patients and the public can bring to health research [15, 17]. Considering context is another key feature of knowledge mobilisation [16], and is also important when working with under-served groups to ensure that activities are tailored to people’s diverse needs and cultures.

**Fig. 1:**
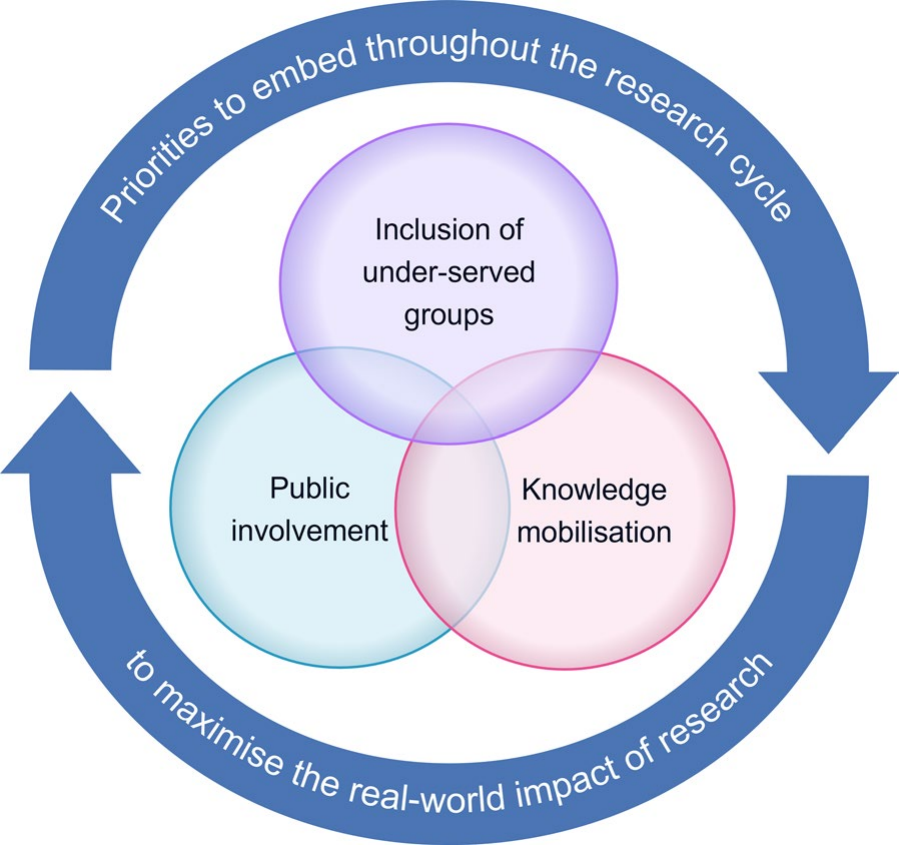
Venn diagram of the concepts of inclusion of under-served groups, public involvement, and knowledge mobilisation. An image description of the diagram is available in Additional file 1

Inclusion of under-served groups, public involvement, and knowledge mobilisation are distinct but inter-related concepts (Fig. 1, Additional File 1). They are distinct in having separate principles, standards, and frameworks to guide them. Key areas of overlap are that they should all be embedded throughout the research cycle to maximise the real-word impact of research, be undertaken in a meaningful rather than tokenistic way, and require adequate resourcing. Although the NIHR emphasises that all three concepts are priorities, they are not always embedded in research practice. For example, in 2023, only 11% of NIHR-funded studies reported that public involvement was used during mobilisation of the study findings for implementation [18]. While there was no breakdown of demographics in the data, it is likely that the involvement of under-served groups in mobilising the study findings was even less, as a previous NIHR survey highlighted limited diversity in public contributors’ backgrounds [19].

While there is currently a focus on these three concepts in UK-based health research, they are not new and many of the principles align with other academic fields and research approaches. The importance of dismantling power imbalances has long been recognised in fields such as disability studies, critical race studies, Indigenous studies, and participatory research approaches [20–22]. Community-Based Participatory Research (CBPR) is a research approach that prioritises meaningful and equitable community participation [6, 22]. CBPR aligns closely with the concepts of inclusion of under-served groups, public involvement, and knowledge mobilisation as it focuses on aspects such as partnership building, power sharing, multidirectional knowledge exchange, and ensuing research has a practical and meaningful benefit for all partners [22]. However, there are important differences between public involvement and participatory research, such as the need to gain informed consent from individuals participating in research projects.

**Fig. 2:**
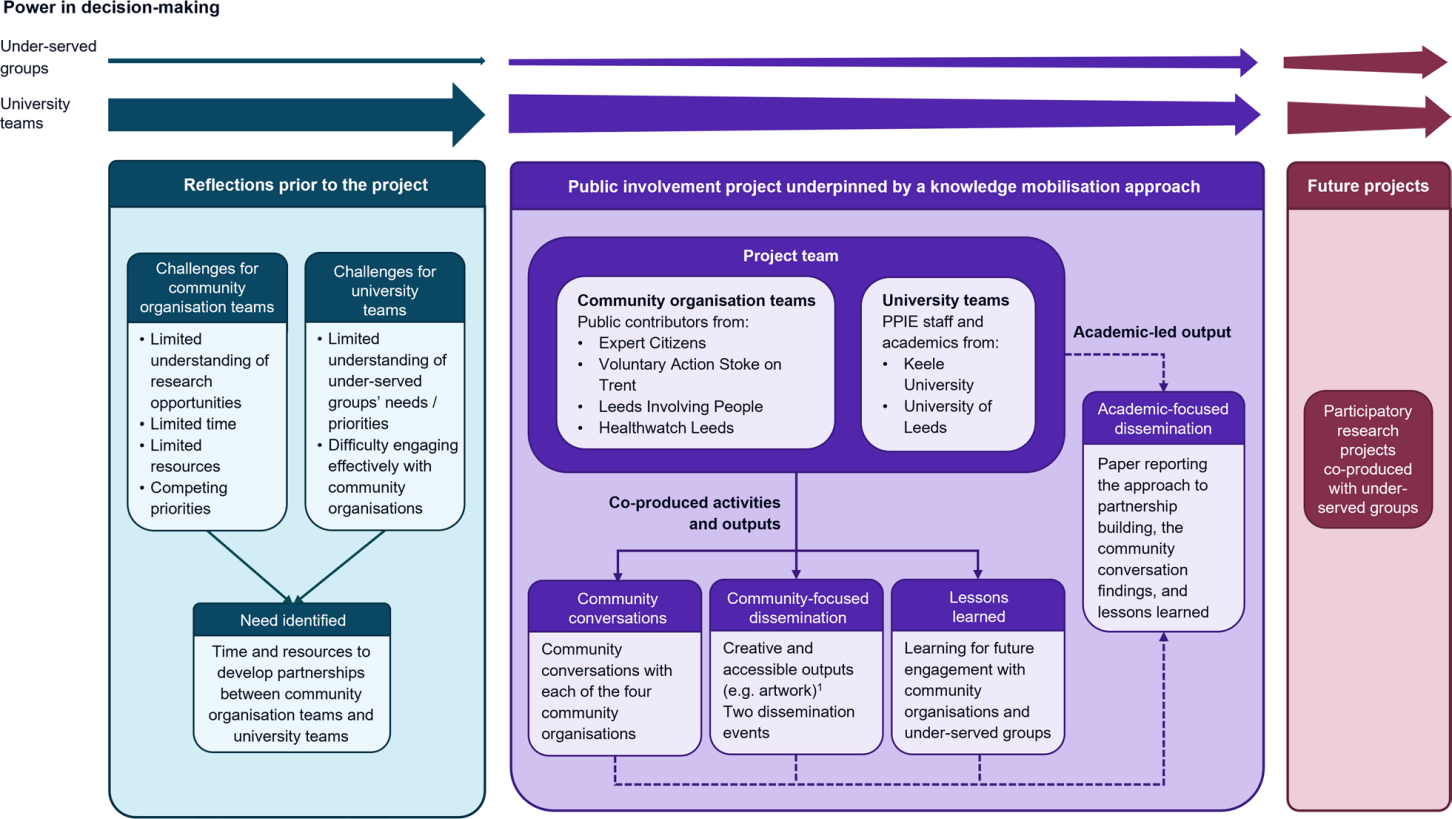
Project strategy. An image description of the strategy is available in Additional file 1. The decisions about which creative and accessible outputs to develop were made jointly by the community organisation teams and university teams. The development of outputs was undertaken by different individuals/groups, including illustrators (one of whom is a Leeds Involving People member), a creative design company, and members of the project team. *PPIE* Patient and Public Involvement and Engagement

This project involved community organisations and university teams coming together to start building partnerships with each other, a need identified through reflections of the community organisation teams and university teams (Fig. 2, Additional File 1). To provide time and resources to start the partnership building, funding was obtained through a novel NIHR funding scheme dedicated to developing innovative, inclusive, and diverse public partnerships [23]. In line with the NIHR’s priorities and short-term nature of the project, it was conceptualised as a public involvement project employing a knowledge mobilisation approach.

The project team included public contributors employed by four community organisations (Expert Citizens, Voluntary Action Stoke on Trent (VAST), Leeds Involving People, and Healthwatch Leeds) and Patient and Public Involvement/Engagement (PPIE) staff and academics from two UK universities (Keele University and the University of Leeds). The community organisations all brought substantial and differing experiences of working with diverse under-served groups as explained in Table 1. The university team members have a range of backgrounds, with a focus on musculoskeletal conditions and self-management. The project team members brought diverse lived experiences to the project, such as being from Asian-heritage communities, having long-term health conditions, being neurodivergent, and having lived experience of combinations of social injustices (e.g. homelessness, drug and alcohol use, and contact with criminal justice systems). The project team’s professional expertise spans numerous areas such as community engagement and involvement (CEI), PPIE, knowledge mobilisation, mixed methods research, and digital interventions. The project team also have extensive expertise in working with underserved groups, informed by their backgrounds.

**Table 1:** Community organisations overview

Organisation	Overview
Expert Citizens	Expert Citizens is a community interest company built by and for people with lived experience of combinations of social injustices, such as homelessness, mental health issues, addiction, and contact with criminal justice systems. Expert Citizens became a community interest company in 2016. Its goal is that systems are led by the people they are designed to serve for the collective interest of society. The Expert Citizens community act as a support network for each other, engaging in team building and promoting healthy lifestyles.
Voluntary Action Stoke on Trent	Established in 1920, Voluntary Action Stoke on Trent is a registered charity, providing specialist services to the VCSE in Stoke-on-Trent and North Staffordshire, supporting them to deliver quality services to local communities. Voluntary Action Stoke on Trent is an advocate on behalf of the VCSE at a strategic level supporting cross-sector work with public sectors partners.
Leeds Involving People	Leeds Involving People is an independent user-led organisation established in 1995. The Leeds Involving People team work with diverse communities and aim to give local people a voice to help improve health, social care, and community services. Their work involves training, supporting, and mentoring their members to enable them to influence decision-makers. They facilitate various user-led groups; offer accessibility training and auditing; and deliver involvement projects with a range of external organisations.
Healthwatch Leeds	Healthwatch Leeds is an independent watchdog organisation established in 2013. It is part of a network of over 150 local Healthwatch organisations throughout England. Its overall purpose is to enable local people to influence health and social care services in Leeds. To do this, Healthwatch Leeds staff and volunteers actively engage with diverse service users, gathering their feedback and supporting them to directly share their views with decision-makers. In addition, Healthwatch Leeds offer information, advice, and signposting to help ensure local people have equitable access to health and social care services.

VCSE, Voluntary, Community and Social Enterprise

The overall project aim was to explore the use of a knowledge mobilisation approach to start building partnerships with under-served groups based on trust and mutual understanding. Key purposes of the project were to identify take-home messages from the project activities and co-produce lessons learned for future engagement with community organisations and under-served groups. The project was intended to start addressing the power imbalances between the under-served groups and university teams to provide a foundation for future participatory research projects (Fig. 2, Additional File 1). By employing a knowledge mobilisation approach with diverse underserved groups at the earliest stage of the research cycle, this project provides a novel example of work at the intersection of inclusion of under-served groups, public involvement, and knowledge mobilisation.

The NIHR advocates sharing experiences of learning from public involvement, including through publications where possible [9, 12]. The purpose of this paper is to report the approach to partnership building, take-home messages from the project activities, and lessons learned for future engagement with community organisations and under-served groups. This contributes to existing literature by providing practical examples and learning for others to draw on when seeking to build inclusive public partnerships.

This was a six-month project with limited resources available. The main priorities during the project were to build relationships and co-produce activities and outputs considered important by the public contributors (Fig. 2, Additional File 1). Writing an academic paper was not a priority to the public contributors, so was undertaken after the project’s official completion. Individuals from all four community organisations involved in the project were offered the opportunity to join the authorship team, with two individuals from Expert Citizens and two individuals from Healthwatch Leeds ultimately deciding to be co-authors. While this approach meant the paper writing was predominantly academically led, the paper is underpinned by the co-produced activities and outputs (Fig. 2, Additional File 1).

## Methods

### Overall approach

This was a public involvement project conducted in line with the NIHR guidance on public involvement in research [24]. The public contributors included the project team members employed by the community organisations, members of the community organisations who were not employed by the community organisations, and additional members of the public. Various approaches can be used for public involvement, including consultation, collaboration, co-production, and user-controlled research [24]. These approaches have different strengths and limitations, so two approaches were combined in this project. These included co-production, in which public contributors jointly share power and responsibilities with researchers throughout an entire project, and consultations, in which researchers ask public contributors for their views and then ensure the public contributors' views have a meaningful impact on their decision making and plans [24].

Overall, a co-production approach was employed to help address potential barriers to inclusive partnership working, such as power and privilege imbalances, limited engagement, and cultural differences. The co-production approach involved public contributors employed by the community organisations jointly sharing power and responsibilities throughout the project as members of the project team. Community organisation members who were not employed by the community organisations were also involved in co-production activities where possible.

As explained below, initial public involvement activities with the community organisation Expert Citizens led to the decision to hold community conversations. The community conversations in this project could be considered a type of consultation, although the project team ensured that co-production principles, such as reciprocity and valuing everyone’s views [25], were embedded throughout the community conversations. For example, all the community conversations involved providing food for the attendees, and the attendees were invited to raise any points they felt were relevant. Further details about the community conversation methods are provided below.

To address the aim, the project also employed a knowledge mobilisation approach. This involved bringing local communities and university teams together to engage in multi-directional dialogues. In line with the principles of knowledge mobilisation, the project team prioritised relationship building, valuing all types of knowledge, and co-creating new knowledge that can make a practical difference in the real world. The project team also aimed to start understanding the contexts of the under-served groups being engaged and tailored the project activities accordingly.

Throughout the project, the project team engaged in a process of continuous critical reflection in line with the co-production approach [25]. This included holding team meetings before and during the project to discuss aspects such as challenges faced, emerging issues related to involving people from under-served groups, and steps that could help the project team to be more inclusive. It also included discussing relevant tools and frameworks, including the Coin Model of Privilege and Critical Allyship [20]. This model uses the metaphor of a coin to highlight how social structures that create unearned privilege (the top of the coin) and oppression (the bottom of the coin) contribute to health inequities. Different systems of inequity are conceptualised as different coins (e.g. racism, ableism, classism, sexism, cisgenderism etc.), hence people may have positions on the top of some coins and bottom of others. Different coins are considered to intersect, creating further forms of privilege and oppression. Critical allyship is conceptualised as an ongoing practice rather than an identify, with a focus on shifting power from people on the top of coins to those on the bottom [20].

The project team aimed to approach this project with the recognition of their positions of privilege and oppression, an awareness of the importance of considering intersectionality, and the intention to shift power from university teams to under-served groups. The project team acknowledge that university teams currently hold more power than under-served groups in this context and, while this project represents an important initial step in addressing this, further work will be required to continue addressing the power imbalances (Fig. 2, Additional File 1). A range of practical steps were taken in this project to help address power imbalances. These included allocating 70% of the total project budget to the community organisations, not using academic titles, holding the community conversations in locations chosen by the public contributors, and ensuring the numbers of public contributors present at the community conversations were greater than the number of university team members present.

### Ethical, inclusion and reporting considerations

Best practice on public involvement was followed as described in the NIHR guidance on public involvement in research [24] and UK Standards for Public Involvement [26]. For example, the project team aimed to ensure the activities were as inclusive and accessible as possible. ‘Inclusion’ was considered as a broad term for ensuring that everyone feels welcome, valued, free to be their authentic selves, and able to contribute as much as they would like to [7, 27, 28]. ‘Accessibility’ was considered as a component of inclusion which is focused on ensuring the needs of disabled people are considered and addressed, so that they can contribute equally without facing disability-related barriers [28–30]. Established resources were drawn on to help guide the EDI considerations. These included the NIHR Research Design Service EDI Toolkit [31] and the NIHR inclusive public involvement guide [9, 12].

Following the community organisations’ standard approaches was key to ensuring that the activities were ethical, inclusive, and accessible, and appropriate safeguarding strategies were in place. For example, the Leeds Involving People community conversation was held in British Sign Language and English at a community centre with a breakout room and mental health first aider available, and the Leeds Involving People standard consent form was used to obtain written informed consent for filming and audio recording. Other examples of steps taken to maximise inclusion and accessibility included holding all the community conversations for home care users in their own homes, facilitating a Healthwatch Leeds community conversation entirely in Cantonese, and developing dissemination materials in a range of accessible formats as described below.

In line with the NIHR guidance [24] and similar projects [13, 32, 33], ethical approval was not sought because this was a public involvement project. The project is reported in line with the GRIPP2 long form for public involvement activities (Additional file 2) [34].

### Community conversations

Numerous methods can be used for public involvement and knowledge mobilisation activities [15, 24]. The method of community conversations was chosen for this project based on initial public involvement activities with Expert Citizens, as they had used that method before and found it to be particularly helpful and engaging for their members. Community conversations involve facilitators holding open discussions with local people in community settings, typically with the aim of stimulating critical thinking, exploring relevant issues, and identifying community-focused solutions [35, 36]. This can help to achieve mutual learning and benefits [35]. The method of community conversations was considered particularly appropriate for this project because it aligns closely with the principles of knowledge mobilisation and can help address power imbalances by giving a voice and ownership of an issue to local communities [37]. In addition, community conversations are more flexible than other similar approaches such as World Cafés [32, 38]. This was a priority to ensure that the public involvement activities could be tailored to suit different communities and a diverse range of people could be involved.

There is no single widely accepted process for conducting community conversations, and various approaches have been used successfully with under-served groups [35, 36, 39–41]. A flexible approach was employed to help ensure the community conversations were tailored to diverse groups’ needs. This involved co-producing separate community conversations with each community organisation (Table 2). During the planning process, university team members broadly outlined what they wanted to achieve, sharing knowledge about health research where appropriate. However, the priority at all stages was to listen to and be guided by the public contributors.

**Table 2:** Community conversations overview

	Expert Citizens (EC)	Voluntary action stoke on Trent (VAST)	Leeds involving people (LIP)	Healthwatch Leeds (HWL)
Planning	Collaboratively led by members of the EC team and Keele University team. The EC team choose four key topics to discuss: 1. Finding out about health 2. Accessibility/language 3. Involving people in research 4. Partnerships The EC team and Keele University team co-produced a framework of questions on the key topics (Additional file 3). All the questions were underpinned by a knowledge mobilisation framework [15].	Led by the VAST team, with input from the Keele University team and local Health Champions representing communities across Stoke-on-Trent. Notes from the EC community conversation were used to help co-produce 19 key statements to use as prompts during the VAST community conversation (Additional file 3). The statements were grouped into four key themes, matching the four EC community conversation key topics.	Led by the LIP team, with University of Leeds team members providing input where required. The LIP team selected two key questions to discuss: 1. What do you think about health research? 2. We need to change the ways we work. What do you think needs to change, and how? The LIP team and University of Leeds team co-produced three slides covering the key questions, additional prompts, and an introduction to the University of Leeds team (Additional file 3).	Led by the HWL team, with the University of Leeds team members providing input where required. The HWL team developed a session plan and notetaking template (Additional file 3). These included eight questions grouped into three areas: 1. Understanding the group’s experiences and needs 2. Removing barriers to getting involved in health research 3. Working together on health research
Format, duration, and reimbursement^1^	Single structured event lasting ~ 3 h. A hot lunch was provided towards the end. Attendees were not given vouchers but some of the project budget was used to purchase items for the Expert Citizens’ facilities and pay for days out for the members.	Single drop-in event lasting ~ 3 h held alongside a local food bank and community café. A hot breakfast and other refreshments were provided throughout the event Additional reimbursement of the attendees was not provided due to the drop-in nature of the event.	Single structured event lasting ~ 4 h. A hot lunch was provided towards the end. Attendees were offered a £25 voucher.	Eight structured one-to-one or group discussions, each lasting approximately 1.5 h. Lunch or refreshments were provided at all the group discussions. Attendees were offered a £20 voucher.
Location	ECs community headquarters, with quiet spaces if needed.	Methodist church.	Local community centre with a breakout room available.	Home care users’ own homes, HWL offices, a community centre, and a Hearing and Sight Loss Centre.
Invitations and adverts	The EC team invited all their community members to attend and encouraged them to discuss the conversation with their peers.	The VAST team sent invites to their health champions from across Stoke-on-Trent to attend and support the event. The church advertised the event through their networks.	The LIP team invited specific LIP members, with the aim of ensuring a diverse range of people attended. Individuals who heard about the event through word of mouth were also welcomed.	The HWL team invited different community groups and individuals to explore barriers experienced by people who have language needs or lived experiences of disability.
Set up	The EC leader welcomed everybody and gave a brief introduction. Conversations were then held around four large tables. Each table was focused on one of the four key topics. Groups rotated round the tables so that all attendees had an opportunity to discuss all the topics. One EC team member and three Keele University team members facilitated the table discussions and took written notes. An illustrator created live visual notes of the conversations.	Chairs were set-up in small groupings to promote conversations and four themed boards were positioned around the room. The boards displayed the key themes and corresponding co-produced statements. Stickers were provided to enable attendees to ‘Agree’ or ‘Disagree’ with the statements. Attendees could add their own comments on sticky notes. Creative activities (e.g., mosaics) were also available. Staff from VAST and the Keele University team moved around the room to support conversations and added comments from attendees to the boards using sticky notes. An illustrator created live visual notes of the conversations.	The University of Leeds team members welcomed everyone and shared the slides. Conversations were then held around large tables. With the attendees’ consent, the discussions were filmed, and some attendees took part in audio recordings. Four LIP staff and two University of Leeds team members facilitated the table discussions and took written notes. A mental health first aider was available. Two BSL interpreters provided BSL interpretation. A LIP member who is an illustrator created artwork during the conversation.	Conversations were held with the following four groups in line with the session plan. Home care users: a HWL staff member facilitated one-to-one conversations with five home care users. Bangladeshi carers group: two HWL staff members facilitated a group conversation in Bengali and English. Chinese group: one HWL staff member facilitated a group conversation in Cantonese, with notes taken in Cantonese then translated into English. Visually impaired group: one HWL and two University of Leeds team members facilitated a group conversation. A creative design company created posters of the conversations once all the conversations had been undertaken.

^1^All the community conversations were relatively informal, with attendees being welcome to come and go as they pleased even when a more structured format was used

*BSL*, British Sign Language; *EC*, Expert Citizens; *HWL*, Healthwatch Leeds; *LIP*, Leeds Involving People; *VAST*, Voluntary Action Stoke on Trent

As highlighted in Table 2, all the community conversations involved holding open discussions about health research in community locations. As with the planning activities, university team members shared knowledge about health research where appropriate, but prioritised listening to the public contributors. Additional activities were used to support the discussions, such as prompt questions, live illustrators, and themed boards that attendees could add ‘Agree’ or ‘Disagree’ stickers to. The knowledge mobilisation framework described by Ward [15] was drawn on to underpin a framework of prompt questions used during the Expert Citizens community conversation (Additional File 3). This framework involves considering why, how, whose, and what type of knowledge is being mobilised [15]. To maximise flexibility, the questions used during the other community conversations were not explicitly underpinned by a knowledge mobilisation framework. During the conversations, research involvement, participation, and engagement activities were all considered given they are important and inter-related components of public partnerships [7]. Demographic information was not collected at most of the community conversations, as the public contributors generally felt that would be detrimental to the relationship building. The approaches used to reimburse attendees varied as described in Table 2.

The community conversations were conducted sequentially between March 2023 and July 2023 in the order listed in Table 2. The findings of the Expert Citizens community conversation informed the planning of the VAST community conversation. The findings of these conversations were not directly used to inform the Leeds Involving People and Healthwatch Leeds community conversations to help ensure the community conversations could be tailored to the attendees. However, the project team members drew on practical learning from the Expert Citizens and VAST community conversations where appropriate. For example, one lesson learned from the Expert Citizens community conversations was that it is helpful for project team members attending community events to dress casually, so the project team members considered that for the Leeds Involving People and Healthwatch Leeds community conversations.

### Reporting the community conversation findings

Key findings from the community conversations were captured through illustrations (visual notes, artwork, and posters) and written notes. The visual notes and artwork for the Expert Citizens, VAST, and Leeds Involving People community conversations were created during the events by live illustrators, one of whom was a Leeds Involving People member (Table 2). This helped to support the conversations, prompt further discussions, and ensure the visual notes and artwork closely reflected the discussions. It also aided the relationship building and helped to address power imbalances. Given the Healthwatch Leeds community conversations were held as eight discussions, having a live illustrator present was not possible so posters were created to bring all the findings together after the conversations. The development of the posters was led by Healthwatch Leeds and undertaken by a creative design company.

Written notes of each community conversation were made by members of the community organisation teams and/or university teams who attended the conversation and then shared with the wider project team. Formal qualitative analysis of the illustrations and written notes was not undertaken because this was a public involvement project. The initial intention was to include a separate summary of each community conversation in the academic paper. However, when reflecting on the initial summaries, the authorship team noticed that key messages were repeated across the conversations, so decided that grouping the findings of all the conversations together would be more appropriate.

The grouping of findings was an inductive process which drew on the framework method [42]. A table was created in Microsoft Word to summarise specific points from the community conversations. This table was used alongside the community conversation illustrations, written notes, and summaries to develop ‘take-home messages’, which align with ‘themes’ in framework analysis [42]. The take-home messages were intentionally descriptive with a low level of interpretation to help ensure they closely reflected the community conversation discussions [43]. Points were considered as important/priorities if the community conversation attendees explicitly highlighted them as important/priorities and/or if they were raised during multiple community conversations.

The main authors involved in developing the summaries, table and/or take-home messages were all university team members (AMA, AM, SRK), but the wider project team reviewed the take-home messages to ensure they genuinely reflected the community conversation discussions. Furthermore, the illustrations and other outputs developed/discussed with the community conversation attendees are included or signposted to in this paper, all of which corroborate the take-home messages.

### Dissemination, feedback, and learning

The dissemination and feedback activities were primarily led by the community organisation teams and community conversation attendees. A range of dissemination materials were developed to share information publicly, including the illustrations discussed above. As detailed further in the results section, creative approaches were used to disseminate and mobilise the key message to help address language, literacy, and accessibility barriers, and provide engaging outputs that could be used by all in varying arenas (e.g., presentations, social media, and notice boards).

Expert Citizens, VAST and Keele University team members co-produced a joint face-to-face dissemination event. Leeds Involving People and University of Leeds team members also co-produced a face-to-face dissemination event. The Healthwatch Leeds team shared the project findings directly with their community conversation attendees, including through a report in written and audio formats, to help ensure the attendees’ language and accessibility needs were fully met. All the dissemination activities were relatively unstructured and relaxed, with the aim of encouraging open and honest discussions.

The community organisation teams highlighted the importance of ensuring any feedback activities were accessible and informal, avoiding approaches such as lengthy feedback forms. In line with that, feedback was mainly obtained through discussions at the dissemination activities and team meetings involving the community organisation staff. Additionally, a brief feedback form was shared at the Keele dissemination event. During the dissemination activities, opportunities for continuing to build partnerships and co-produce future projects were explored.

At the end of the project, the project team held further team meetings to co-produce lessons learned for future engagement with community organisations and under-served groups. The project team aimed to ensure that the lessons learned captured the key findings of the community conversations as well as their personal reflections developed through the process of continuous critical reflection described above.

## Results

### Community conversations overview

All the community conversations were engaging events with diverse attendees (Table 3).

**Table 3:** Community conversation attendees

Community conversation	Overview of attendees
1. Expert Citizens	Approximately 20 Expert Citizens members, including people with experience of combinations of social injustices such as homelessness, contact with criminal justice systems, abuse, mental health, and addiction issues.
2. Voluntary Action Stoke on Trent	Approximately 45 people^1^, including volunteers from the methodist church who provided refreshments, Health Champions linked to communities across Stoke-on-Trent, local people accessing the foodbank, and other members of the public.
3. Leeds Involving People	22 Leeds Involving People members, including people from minority ethnic groups, people from the Deaf and hard of hearing community, neurodivergent people, and people with various physical and mental health conditions.
4. Healthwatch Leeds	27 people in total, including five home care users, seven people from the Bangladeshi carers group, eight people from the Chinese group, and seven people from the visually impaired group.

^1^ Some of the people who attended the Voluntary Action Stoke on Trent community conversation wished to use the food bank and leave without joining the conversations or activities

Attendees’ experiences and understanding of health research varied widely. Many attendees at the Expert Citizens and Leeds Involving People community conversations reported experience of participating in research. However, when the Leeds Involving People community conversation attendees were asked to explain more about the research, they tended to describe service redesign and PPIE activities led by healthcare bodies such as a local National Health Service (NHS) trust and Integrated Care Board. Additionally, some Expert Citizens and Leeds Involving People community conversation attendees reported not knowing how to get involved in research.

Most Healthwatch Leeds community conversation attendees did not have experience of participating in health research because they were unaware of it, had not been invited, did not meet the criteria, or encountered accessibility problems. The drop-in nature of the VAST community conversation meant attendees’ previous experiences of research were not explored in depth. However, eight attendees added ‘Agree’ stickers for the statement ‘I don’t know where or how I can get involved in research or what type of research I can be a part of’, while six attendees added ‘Disagree’ stickers for the same statement.

Most of the discussions did not distinguish between involvement, participation, and/or engagement in health research as aspects such as why, how, and where research-related activities are carried out appeared to be more meaningful to the attendees. To reflect this, the following sections generally use the phrase ‘contribute to’ rather than ‘be involved in’, ‘participate in’ or ‘engage in’.

### Recognising the appetite for change

Attendees at all the community conversations expressed a strong appetite for change in healthcare and health research, and a desire to contribute to health research. In many instances, attendees’ appetite for change appeared to be driven by negative experiences of current health services. For example, numerous attendees reported struggling to access General Practitioner (GP) appointments or feeling that mental health service provision was lacking. Other attendees were much more positive about current health services, with some attendees emphasising the value of the NHS.

Across all the organisations’ community conversations, attendees identified multiple barriers that limit their potential to contribute to research. They also made numerous suggestions related to addressing the barriers and building inclusive public partnerships. These barriers and suggestions were collated into three inter-related take-home messages to consider when working with under-served groups in the future:

1. Ensure relevance, appreciation, and trust
2. Prioritise language and accessibility needs
3. Maximise flexibility in all research-related activities

**Fig. 3:**
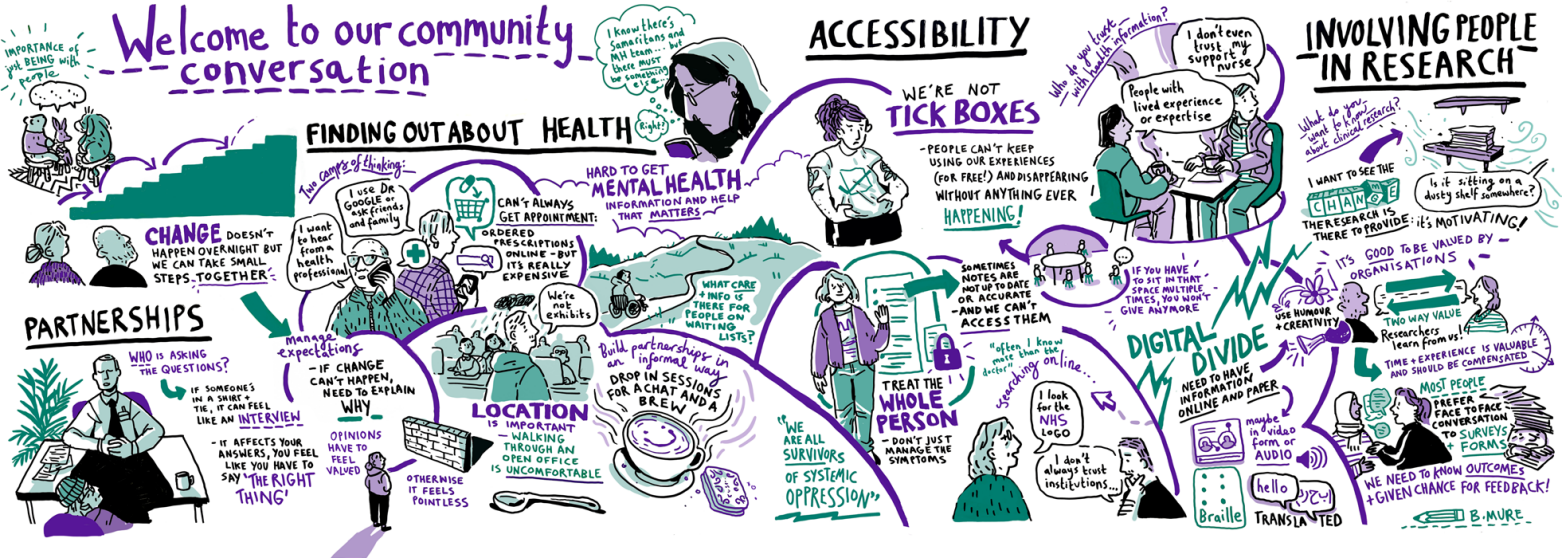
Expert Citizens community conversation visual notes. An image description of the visual notes is available in Additional file 1

**Fig. 4:**
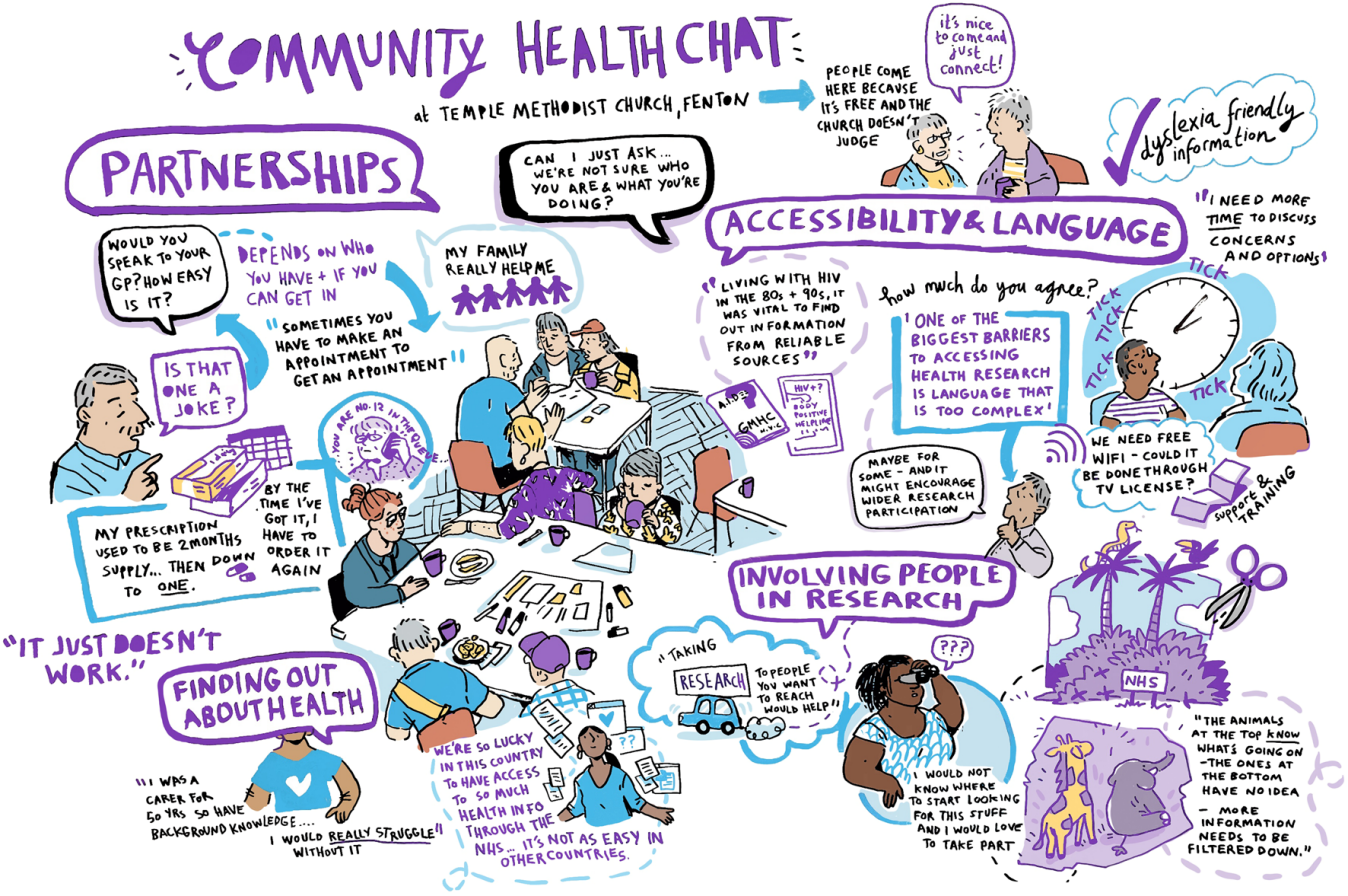
Voluntary Action Stoke on Trent community conversation visual notes. An image description of the visual notes is available in Additional file 1

**Fig. 5:**
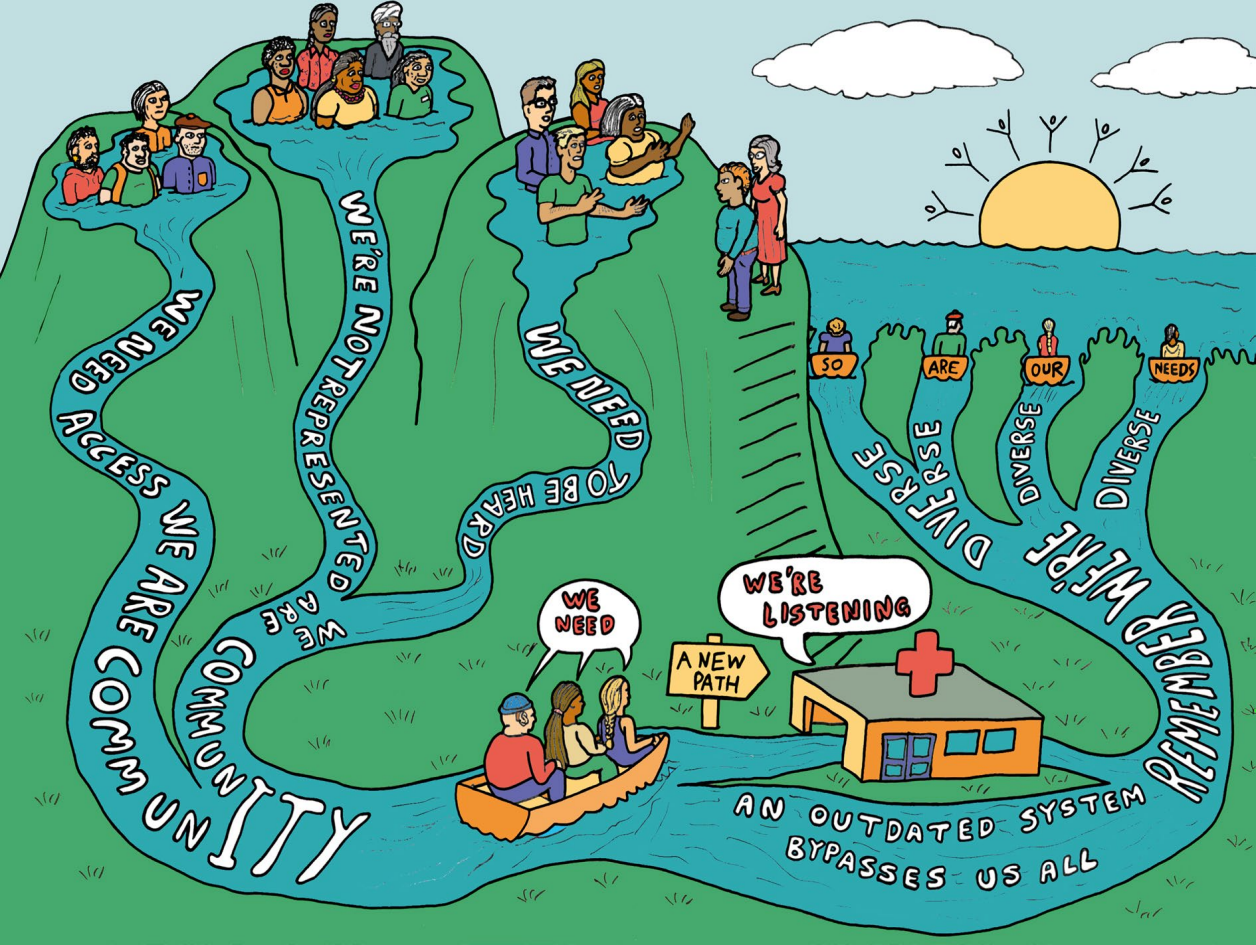
Leeds Involving People community conversation artwork. An image description of the artwork is available in Additional file 1

**Fig. 6:**
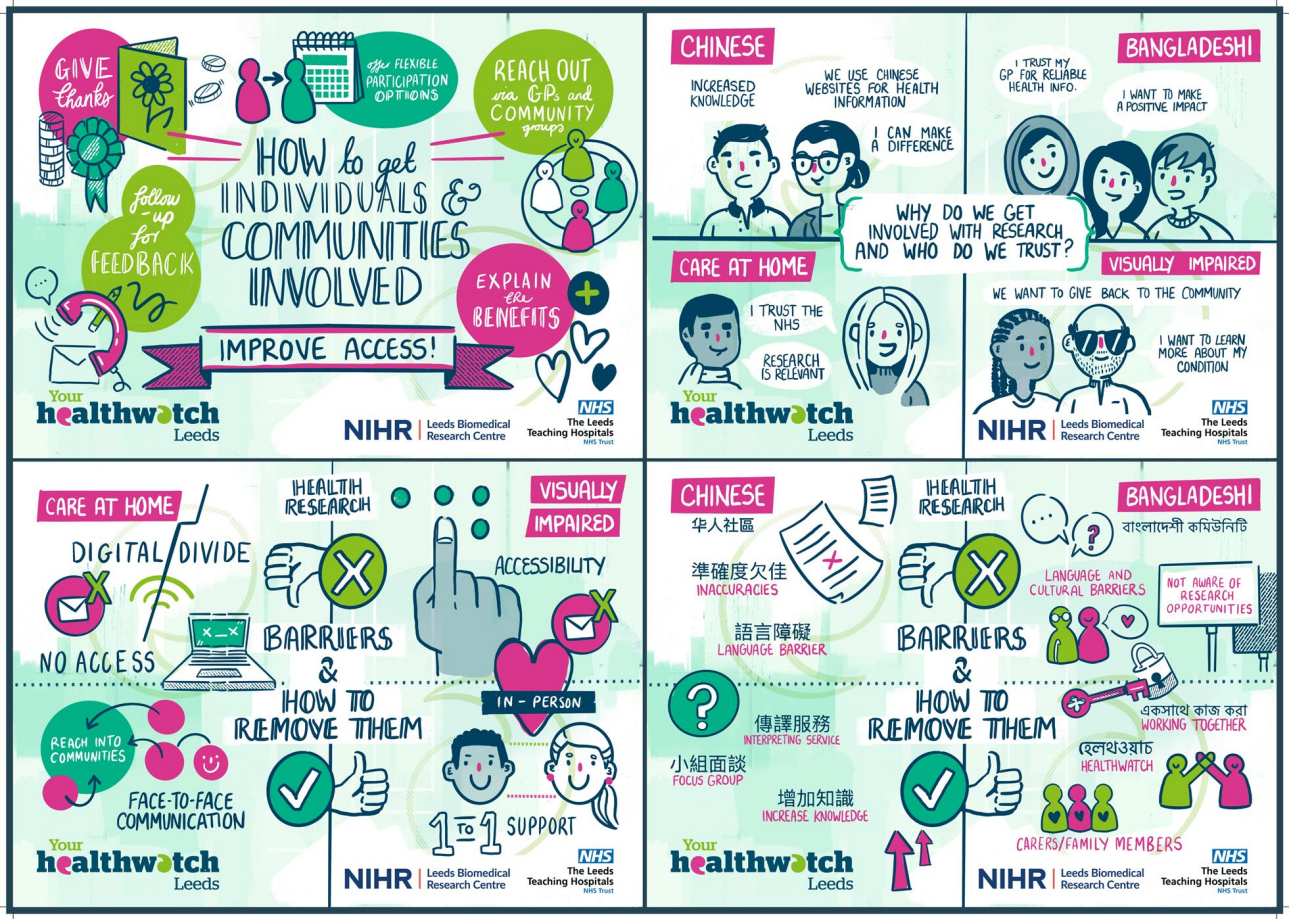
Healthwatch Leeds community conversations combined posters. An image description of the posters is available in Additional file 1

Each take-home message is discussed below and supported by the visual notes, artwork, and posters from the community conversations (Figs. 3, 4, 5, and 6, Additional file 1).

### Ensure relevance, appreciation, and trust

Attendees from all the organisations’ community conversations identified various factors related to relevance, appreciation and trust that would encourage them to contribute to health research. These included the research being personally relevant to them; understanding the potential benefits of the research; feeling like their contributions would make a positive difference for others; feeling their input was valued, respected, and appreciated; and trusting the research teams.

Some attendees felt those considerations are not addressed at present. For example, Leeds Involving People community conversation attendees felt current research does not focus on what matters most to local communities. A key concern was that health research appears to be financially driven by large pharmaceutical companies, with a focus on benefitting Western populations. Autistic and Deaf/hard of hearing attendees were also particularly concerned about genetic research and the risk of eugenics. Another reported issue was lack of aftercare for research participants, particularly if they had been given new information about their health that they did not know how to follow up on. Numerous attendees were unimpressed by not receiving feedback about research they had contributed to. Similarly, limited sustainability of research partnerships was considered problematic and appeared to contribute to mistrust of researchers.

Key suggestions for addressing these issues included enabling people with relevant lived experience to plan and lead research; organising research directly with and through community organisations; and developing trusted and respectful relationships with researchers over time. Expert Citizens community conversation attendees suggested it would be particularly helpful to build partnerships with researchers in an informal way through activities such as drop-in sessions where people can have a hot drink and chat. Some attendees also emphasised it is important for researchers not to make judgements/assumptions and ensure they recognise diversity and disabilities.

Many attendees highlighted it is vital for researchers to be clear about why the research is being carried out and what impact it will have on the NHS and their community. Attendees generally felt it is essential to pay/compensate everyone fairly for their time and let people know about that in advance. In addition, providing timely and accessible feedback was considered key to building trust and demonstrating that people’s views and experiences had been respected and included.

### Prioritise language and accessibility needs

Across all the organisations’ community conversations, prioritising language and accessibility needs was identified as an essential requirement for enabling people to contribute to health research. For example, the Bangladeshi carers and Chinese groups reported language is the biggest barrier they face to accessing health information and research. Similarly, complex language was highlighted as a key barrier by attendees at all the other community conversations, with some attendees highlighting too much information is scary. Attendees also identified a range of accessibility barriers for disabled people, with many sharing examples of their accessibility needs not being met when attempting to access healthcare or research. Key issues included problems with wheelchair transport, lack of sign language interpretation, and failure to provide information in large print or audio formats.

Attendees from all the organisations’ community conversations made various suggestions for addressing language and accessibility barriers. These included providing interpreters and ensuring information is brief, easily understandable, and illustrated with graphics. Some attendees felt offering information in different languages should be the norm, rather than something people need to fight for. Similarly, attendees felt addressing people’s accessibility needs should be planned from the outset and not seen as a problem. Suggestions for making research more accessible included offering information in accessible formats (e.g., audio rather than print), providing sign language interpreters, ensuring transport and buildings are accessible for wheelchair users, and travelling to people with mobility or visual impairments rather than expecting them to come to researchers. Providing disability awareness raising/accessibility training for research teams was highlighted as a priority. Undertaking research on accessibility was also suggested. Another suggestion was to ensure that people can be accompanied by an interpreter/carer, with some attendees suggesting that carers should be allowed to participate in research in their own right.

This relates to take-home message 1 discussed above, as ensuring people’s language and accessibility needs are met appears to be essential for ensuring relevance, appreciation, and trust. For example, providing information in understandable and accessible formats, such as the dissemination materials detailed below, is important for enabling people to understand how research is relevant to them and building trust in research teams.

### Maximise flexibility in all research-related activities

The importance of maximising flexibility in all research-related activities was evident at all the organisations’ community conversations. This includes providing flexibility in aspects such as how to share research opportunities, how and when people can get involved in research, how people are paid/compensated for their time, and how research findings are disseminated. Various suggestions were made about how to share research opportunities, including researchers going to community groups, having a research hub that moves around different locations, and sharing information about research through trusted people/sources. Many attendees at the Expert Citizens community conversation reported trusting their peers at the community centre, not researchers or healthcare professionals. Trusted sources identified by attendees at the other community conversations included NHS staff, community organisations, faith groups, people’s younger relatives, and Chinese websites.

Many attendees expressed a preference for contributing to research through face-to-face discussions, particularly if the discussions are held in a familiar environment such as a community centre or local church. In contrast, the home care attendees generally liked the idea of contributing to health research through online group sessions with buddies, as they felt buddies would provide psychological support and ensure accessibility. They also highlighted that online groups sessions would allow for comfortable sharing of ideas in their own familiar environment.

A number of attendees highlighted the importance of offering flexibility with the timing of research activities, for example to avoid people needing to travel early in the morning and allow people to be accompanied by unpaid family carers, who may have other commitments during standard office hours. Flexibility with timescales was considered particularly important for people with mental health challenges. In addition, attendees highlighted it is important to provide extra time if needed for activities such as discussing questions and completing questionnaires. Some attendees felt it would be helpful to offer other forms of support for certain activities. For example, VAST community conversation attendees suggested free Wi-Fi and digital skills training could help people to access health research information on the Internet. Furthermore, numerous attendees emphasised the importance of offering information in both digital and non-digital formats.

Attendees made various suggestions related to being paid/compensated for their time, including covering expenses and carer costs, receiving vouchers, and being given a buffet lunch. A wide range of suggestions were also made regarding preferred formats for receiving feedback on research, such as videos with audio descriptions, infographics, CDs, podcasts, face-to-face or online meetings, and telephone conversations, with opportunities to ask questions. Maximising flexibility through approaches such as offering information in a range of accessible formats is key to ensuring relevance, appreciation, and trust; and addressing people’s language and accessibility needs. Therefore, this take-home message is inter-related with those discussed above.

### Dissemination, feedback, and learning

The dissemination materials developed to share publicly, including with the community conversation attendees, include the visual notes, artwork and posters from the community conversations developed by the individuals/groups described in the methods (Figs. 3, 4, 5, and 6, Additional File 1); a blog of the Expert Citizens community conversation developed by a Keele University team member [44]; a poster incorporating the Leeds Involving People community conversation artwork developed by University of Leeds team members (Additional file 4); a flyer of the Leeds community conversations developed by University of Leeds team members (Additional file 5), and a report of the Healthwatch Leeds community conversation in written and audio formats developed by Healthwatch Leeds and a creative design company [45]. Attendees at the dissemination activities were generally positive about the dissemination materials, commenting on aspects such as the colourful artwork. A few suggestions for improving the materials were made. These included increasing the colour contrast on the Leeds Involving People community conversation poster and explicitly mentioning the ‘Deaf community’ and ‘Wellbeing group’ on the Leeds community conversations flyer.

Feedback about the community conversations and dissemination activities was also largely positive. For example, attendees reported feeling very involved, appreciated having a shared purpose and values, and welcomed the opportunity to join a dissemination event. Attendees from Expert Citizens also reported that the group size worked well, as having more community members than university team members helped to address power dynamics. Another aspect that was very positively received was facilitating the Chinese group’s community conversation and feedback session entirely in Cantonese, as that enabled them to fully understand what was being discussed. A notable piece of negative feedback was that some attendees disliked seeing a researcher take “private notes” as they felt uncomfortable about not being able to see what was being written. This appeared to undermine their trust in the university team. Having large pieces of paper and sticky notes for writing down key points was much more positively received. While the themed boards with ‘Agree’ and ‘Disagree’ stickers at the VAST community conversation were helpful for encouraging conversations, the Expert Citizens team suggested not using the terms ‘Agree’ and ‘Disagree’ as that could be interpreted as some communities being in the wrong.

The joint dissemination event with Expert Citizens and VAST was useful for building relationships between the two community organisations themselves, as well as with the Keele University team. The relatively unstructured and relaxed nature of the dissemination activities appeared to work well, with attendees being open to sharing positive and critical feedback. The dissemination activities also enabled issues raised at the community conversations to be explored in more depth. For example, the Leeds Involving People dissemination event attendees recommended building in time for reflective practice and made additional suggestions about how they would like to be paid/compensated for their time. These included being offered a menu of options such as training courses (e.g., in first aid/mental health first aid), certificates, charity donations, a travel card and gas/electricity meter top-ups.

Attendees at the dissemination events said they are keen to contribute to future research opportunities. In addition, all the community organisation teams stated they would like to continue building partnerships with the university teams, including through being involved in activities organised by the university teams and/or through leading future work. A range of potential next steps for continuing this project’s partnership building were discussed, such as developing accessibility training for researchers and organising ‘Ask the Researcher’ engagement events to enable researchers to discuss their research with local communities. Since the project’s completion, the University of Leeds team have undertaken further public involvement activities with Leeds Involving People and Healthwatch Leeds to plan a grant application for a participatory research project involving the development of accessibility training for researchers and are aiming to carry out engagement events with both community organisations in the future. In addition, this project’s findings have fed into the development of ‘The Leeds Approach to engaging with communities for research’ led by the team’s Community Co-ordinator and endorsed by the Leeds People’s Voices Partnership (PVP) [46]. The Keele University team recently hosted a community celebration event to help foster an ongoing relationship with Expert Citizens and VAST.

Table 4 presents the lessons learned for future engagement with community organisations and under-served groups, which capture key findings of the community conversations and the project team’s personal reflections.

**Table 4:** Co-produced lessons learned for future engagement with community organisations and under-served groups

Area	Lessons learned^1^
General principles	Co-produce research activities with community organisations and people with relevant lived experience.
	Consider combining different approaches to public involvement, such as co-production and consultations.
	Consider drawing on principles of knowledge mobilisation during public involvement and engagement activities.
	Ensure people with relevant lived experience are actively involved in all research activities, including conceiving research ideas, and leading planning, and delivering research activities.
	Take time to build trusting relationships wherever possible e.g., by providing activities such as drop-in sessions where people can have a hot drink and chat.
	Aim for everyone involved in research activities to have a shared purpose and values, including through outlining expectations at the start of activities and building in time for reflective activities.
	Ask people what their needs and preferences are regarding areas such as language, accessibility, terminology etc., rather than making judgements or assumptions.
	Ensure any research-related information is brief, easily understandable, and illustrated with graphics, and translated/accessible formats are available for people who need them.
	Ensure research teams are aware of relevant sensitivities of the communities they are working with, including factors that may contribute to mistrust of health research.
	Provide research teams with relevant training, including disability awareness/accessibility training.
	Ensure research team members have access to appropriate support when needed, such as peer support at reflective practice sessions and professional support via counselling services.
Planning	Co-produce involvement activities and research plans with community organisations and people with relevant lived experience.
	Consider holding collaborative planning meetings if more than one community organisation is involved in the project.
	Plan how to address language needs, including through translation and interpreting, and aim to offer research activities that are entirely in attendees’ own language if possible.
	Plan how to address accessibility needs, including needs related to the activity timing, travel, location, and communication.
	Plan for people to be accompanied by a carer/interpreter if needed.
	Consider planning separate activities that are tailored specifically to the needs of certain groups.
	Consider how many people to invite to group activities, aiming to have more community members than research team members if possible.
	Plan a variety of ways for people to contribute to research activities, including through face-to-face activities and online activities.
	Plan support for people who want to join online activities, such as by offering buddies, free Wi-Fi, and/or digital skills training.
	Plan how to pay/compensate people for their time, including by covering all expenses and offering different recognition options, and ensure it is clear in advance how people will be paid/compensated.
Delivery	Co-deliver research activities with community organisations and people with relevant lived experience.
	Share research opportunities through a variety of approaches, including by going to community groups and sharing information through trusted sources.
	Deliver face-to-face activities in people’s familiar environments e.g., community centres, places of worship, and people’s own homes.
	Dress casually when meeting with community groups.
	Enable carers to contribute directly to research activities if appropriate.
	Ask attendees about their preferences for how notes from discussions are recorded, including whether they want them to be visible.
	Consider having an illustrator present to capture conversations visually.
	Consider having interactive activities such as themed boards and ensure that all the communities involved are happy with any activities used.
	Consider providing food to provide informal opportunities for conversations to continue.
Dissemination and feedback	Co-produce dissemination/feedback materials and activities with community organisations and people with relevant lived experience.
	Consider holding joint dissemination events when working with more than one community organisation.
	Ensure research participants are given appropriate follow up if needed, particularly if they are given new health information.
	Ensure feedback about research is provided in a timely manner, even if that means sharing draft materials.
	Show how people’s views and contributions have been respected and included in research.
	Develop dissemination materials in a range of accessible and engaging formats, with opportunities to ask questions.
	Consider organising relatively unstructured and relaxed dissemination and feedback activities to help build relationships and gain further feedback from community organisations and people with relevant lived experience.
	Be realistic about what suggestions and ideas are feasible to address and which may be limited by restrictions such as funder and university procedures.

^1^ ‘Research activities’ refers to research involvement, participation, and engagement activities

*NHS*, National Health Service

## Discussion

### Key findings

This co-produced public involvement project has demonstrated the value of employing a knowledge mobilisation approach to start building partnerships with under-served groups. Employing a flexible approach in which all parties’ knowledge was valued gave people from under-served groups a platform to share their opinions and be heard. The novel NIHR grant that this project was funded through encouraged the use of inclusive knowledge mobilisation approaches from the start of the research cycle, whereas typically knowledge mobilisation activities are conducted at the end of the project. Knowledge mobilisation is a relational and context-dependent process [16], which aligned well with the shared focus on building trusting relationships with under-served groups with widely differing needs and perspectives.

The diversity of groups engaged was a key strength of this project as it enabled cross-context learning to be gained. The take-home messages identified in this project reinforce priorities highlighted by other guidance and research approaches. Novel contributions of this project include the practical examples and co-created learning related to inclusion and partnership working in health research. The co-creation of learning was achieved through a series of many meetings and community conversations, rather than being a single event, and reflects an authentic blend of different types of knowledge. By reporting the project in detail, this paper provides a worked example of how to embed the concepts of inclusion of under-served groups, public involvement, and knowledge mobilisation at the earliest stages of the research cycle.

### Relationship to existing guidance and literature

Many of the lessons learned reinforce recommendations in the NIHR inclusive public involvement guide [9, 12] and CHICO guidance [13] discussed in the background section. For example, the NIHR guide and CHICO guidance highlight the importance of flexibility. This project’s findings reinforce this and provide practical examples of how to maximise flexibility, for example by offering a menu of options for compensating people for their time. The importance of prioritising accessibility for disabled people is a key finding of this project that is not emphasised in the NIHR inclusive public involvement guide [9, 12] or CHICO guidance [13]. This is likely to be because this project specifically involved groups with accessibility needs, including Deaf/hard of hearing people, people with visual impairments, and home care users.

Initiatives to improve inclusion in health research relatively rarely focus on disabled people, despite evidence demonstrating that disabled people experience substantial health inequities and are often unnecessarily excluded from health research [47, 48]. However, some practical resources on improving the accessibility of research are available. For example, Rios et al. [49] propose an approach to accessible design of mainstream quantitative epidemiological, public health, and outcomes research with three levels – universal design, accommodations, and modifications; the Multi-Regional Clinical Trials Center Accessibility by Design Toolkit provide key points and tools for improving the inclusion of disabled people in clinical research [50]; and Bailie et al. [48] have provided a call to action for more disability-inclusive health policy and systems research, which includes advice on how to make online focus groups more accessible and inclusive.

This project’s findings also highlight that improving inclusion of disabled people in health research is a complex issue, which cannot be resolved simply by addressing accessibility barriers. Instead, additional multi-faceted strategies are required to address barriers such as mistrust in health research. Mistrust appeared to be a key barrier to inclusion in health research for many of the under-served groups in this project, aligning with previous research [4, 51, 52]. Practical steps that can help to build trust identified in this project include organising informal activities, providing timely accessible feedback, and asking people’s preferences about how notes are recorded. Valuing the community organisation teams’ knowledge, skills, and existing relationships with under-served groups, rather than viewing them simply as gatekeepers, also helped to build trust. This aligns with previous research highlighting the benefits of employing an ‘asset-based approach’ when working with community organisations [52].

Building trust is recognised as key to participatory research approaches and is closely interlinked with power sharing [21, 22]. In their discussion of CBPR, Rhodes et al. [22] suggest that sharing financial resources is an indicator of power sharing. As highlighted above, 70% of the total budget for this project was allocated to the community organisations. The university teams took extensive steps to change policies within their institutions to permit this with the support of the NIHR. This approach enabled the community organisations to reimburse the community conversation attendees in whichever way(s) they felt were most appropriate, leading to a range of approaches being used (Table 3). A disadvantage of this approach was that it meant some public contributors were reimbursed in different ways despite being involved in similar activities. For future projects involving multiple community organisations, it may be helpful to explicitly discuss reimbursement as part of collaborative planning meetings to ensure that any differences are considered fair and justified.

Holding collaborative planning meetings could also help avoid the use of activities that are considered appropriate by one community but not another, as was the case for the themed board ‘Agree’ and ‘Disagree’ stickers in this project. In addition, it may be worth exploring ways to allow and manage disagreements that arise during partnership working, as providing an open space for disagreements can help to achieve meaningful co-production [53].

### Key reflections

Reflexivity is recognised as an important practice for identifying and addressing power imbalances [20, 21]. In this project, the project team found it helpful to hold their own reflective sessions shortly after the community conversations and associated activities. These provided an opportunity to debrief and discuss practical and emotional challenges that had arisen. Some of the topics discussed with the community conversation attendees were highly emotive and potentially triggering. In addition, some project team members had to consider if/how to make personal disclosures about their own lived experiences. This demonstrates the importance of ensuring that project team members have access to appropriate support when needed, such as peer support at reflective practice sessions and professional support via counselling services. Addressing the project team’s training needs was also identified as important in this project. This could include community members providing mentoring to university team members and vice versa, as well as more formal training.

A key reflection of the project team was that it is important for researchers to be aware of relevant sensitivities of the communities they are working with, including factors that may contribute to mistrust of health research. The concern that research is focused on benefitting Western populations identified in this project aligns with the growing body of literature on decolonalising global health and an NIHR survey showing a disproportionately low level of racial and ethnic diversity in public involvement [19, 54]. Recent consultations undertaken by the NIHR’s Race Equality Public Action Group (REPAG) have highlighted the ongoing impact of racial injustices and cultural incompetencies in relation to health and care research, including *‘enduring issues of harm, betrayal, trauma and loss of confidence’* [55, 56]. Key NIHR initiatives intended to help improve racial equality include the development of an INCLUDE Ethnicity Framework and Race Equality Framework [8, 55]. While such tools and frameworks can provide the foundations for building trust and inclusion, their impact will not be fully realised until their uptake is widespread and inclusive approaches become embedded into routine research practice.

### Benefits and challenges of the community conversations

As well as prioritising co-production principles throughout the project, a more consultative approach to public involvement was employed by holding community conversations. This facilitated the involvement of a more diverse range of public contributors than would have been possible with a purely co-production approach (Table 3). The NIHR defines consultations as a process in which research teams ask public contributors for their views and then use the public contributors' views to inform their decision making [24]. This project’s findings suggest the definition of consultations could be broadened to emphasise that consultations should not be considered a one-way process in which only research teams benefits, and certain co-production principles such as reciprocity can still be embedded within consultatory approaches.

Holding community conversations was found to be a valuable method for knowledge mobilisation due to being simple, inclusive, and flexible. The importance of flexibility was evident from the diversity of community conversation formats chosen by the community organisations. The Expert Citizens and Leeds Involving People teams chose to hold events for their members, so many attendees knew each other. This appeared to encourage open and honest conversations and helped address power dynamics with the university team members. The VAST team opted for a public drop-in event, helping to reach people who might not have joined a more structured event. Separate events for specific groups were organised by the Healthwatch Leeds team. This ensured the groups’ language and accessibility needs were met, which appeared to be particularly valued. Correspondingly, the Leeds Involving People team suggested holding separate sessions for the Deaf and hard of hearing community in the future to allow more time for explaining unfamiliar concepts, such as research, in sign language. Another benefit of the community conversations was that they were undertaken as a public involvement activity, rather than a formal research project. This provided greater flexibility and removed various processes that can be off-putting and intimidating, such as the need for consent, recordings/formal note taking etc.

Having illustrators attend the community conversations proved valuable as the developing visual notes/artwork provided a point of interest and encouraged conversations. This corresponds with previous research highlighting that using creative approaches and collaboratively developing outputs in real time can help stimulate discussions [52, 53]. It is also important to recognise that illustrators can be costly, and illustrations may not be accessible to some groups (e.g., people with visual impairments). This project provides practical examples of other approaches for stimulating discussions, such as the themed boards.

### Limitations

While this project involved a wide range of public contributors, only individuals employed by the community organisations were members of the project team. These individuals were therefore in positions of higher power than other public contributors, but that does not negate the valuable professional and lived experiences and insights they brought to the project. As highlighted in the background and methods, this paper was written after the project’s completion with four public co-authors. Discussing the paper’s findings with the community conversation attendees and including additional public co-authors would have boosted the credibility of this paper. Prioritising the paper writing would have gone against this project’s focus on relationship building and prioritising outputs considered important by the public contributors. Furthermore, including additional public contributors without adequate time and resources would likely have led to them being included in a tokenistic way, potentially undermining the aim of this project. Tensions between communities’ priorities and academic working practices/expectations are a recognised challenge of participatory research approaches [21]. While there is no easy solution, an important first step is to acknowledge the tensions so that they can be considered as the partnerships develop [21].

The priority throughout the project was to listen to and be guided by the public contributors. This meant there was relatively little focus on the university team members sharing information about health research. While this could be considered a limitation from a knowledge mobilisation perspective, it had many benefits in terms of relationship building and enabling the public contributors to have a platform to share their views. Furthermore, the approach employed still enabled the public contributors and university team members to work together to co-create new knowledge. The definition of under-served groups is highly context-specific [3, 4]. This project did not involve various groups that are commonly under-served by health research, such as people living in remote areas and people who lack capacity to consent for themselves; therefore, the findings are not necessarily applicable to other contexts and under-served groups.

Another important limitation of the project was its short duration. This meant it was only possible to start building relationships and it will be necessary to seek further funding/other opportunities to develop the partnerships further and explore how best to support the longevity and sustainability of these partnerships. Keeping this project’s momentum going is a priority for building trust. However, that may be challenging due to systemic factors beyond the direct control of community organisations and university teams, such as lengthy research funding application processes, the time-limited nature of research funding, and the pressures that community organisations often face regarding funding and priorities. Individuals from different organisations and groups may sometimes be able to build relationships outside of research projects, for example through voluntary and social activities. Yet that is often not feasible or appropriate due to factors such as people’s other commitments and the need to maintain professional boundaries in some circumstances. Systemic changes are therefore needed to provide capacity for building and maintaining trusting and reciprocal relationships.

### Implications for future projects

The project team are using the locally co-created knowledge in this project to complement more formally developed guidance on public involvement, such as the NIHR inclusive public involvement guide [9, 12]. This project’s practical examples, findings, and lessons learned could also be useful for other public partnership projects. While the take-home messages are applicable to all research-related activities, maximising flexibility is more challenging to embed in participation activities due to grant applications requiring detailed research plans to be pre-specified and the fixed nature of research protocols. However, flexibility underpins many of the suggested strategies for ensuring relevance, appreciation, and trust; and addressing people’s language and accessibility needs. This demonstrates the importance of involving diverse public contributors early to ensure that sufficient flexibility is built into grant applications and protocols. In addition, there is a need for systemic changes, such as weighting of research funding schemes towards projects that demonstrate a stronger focus on integrating inclusion of under-served groups, public involvement, and knowledge mobilisation throughout the research cycle, with flexibility in the methods and approaches employed.

## Conclusions

This project employed a knowledge mobilisation approach to facilitate multi-directional dialogues between local communities and university teams. The co-produced approach enabled the beginnings of relationship building with under-served groups, resulting in the generation and mobilisation of knowledge that could make a practical difference in the real world. The take-home messages and co-produced lessons learned provide practical suggestions of how to promote inclusion in research involvement, participation, and engagement activities. Maximising flexibility appears to be particularly important for all activities. Addressing the training and support needs of research teams is also a priority to ensure research teams are aware of relevant sensitivities of the communities they are working with and support them to manage any practical and emotional challenges they encounter. This project has provided a firm foundation to build upon, with all the groups expressing a strong appetite for change and desire to continue working together. However, building sustainable, trusting, and inclusive public partnerships arguably requires systemic changes, such as long-term investment in partnership building.

## Supplementary Information

Additional file 1

Additional file 2

Additional file 3

Additional file 4

Additional file 5

## Abbreviations

CHICO: CHecklist for Inclusive COmmunity involvement in health research
EDI: Equality, Diversity and Inclusion
INCLUDE: Innovations in Clinical Trial Design and Delivery for the Under-served
NHS: National Health Service
NIHR: National Institute for Health and Care Research
PPIE: Patient and Public Involvement/Engagement
UK: United Kingdom
VAST: Voluntary Action Stoke on Trent

## Declarations

### Availability of data and materials

No datasets were generated or analysed during the current study.
