# Supplementary material for: Building trust and inclusion with under-served groups: a public involvement project employing a knowledge mobilisation approach
Source: Res Involv Engagem. 2024 Nov 11;10:122. doi: 10.1186/s40900-024-00647-2 (PMC11555807; doi:10.1186/s40900-024-00647-2)
Supplement: Supplementary file 1 — Additional file 1 [file 40900_2024_647_MOESM1_ESM.docx]

Building trust and inclusion with under-served groups: a public involvement project employing a knowledge mobilisation approach

Additional file 1: Image descriptions of the figures

# Image description of Figure 1: Venn diagram of the concepts of inclusion of under-served groups, public involvement, and knowledge mobilisation

Venn diagram with three overlapping circles labelled ‘Inclusion of under-served groups’, ‘Public involvement’, and ‘Knowledge mobilisation’.

The circles are surrounded by a ring of two arrows representing the research cycle, which state ‘Priorities to embed throughout the research cycle to maximise the real-world impact of research’.

# Image description of Figure 2: Project strategy

Summary of the project strategy with three main boxes representing three stages – reflections prior to the project, the project itself, and future projects. Arrows above the boxes depict how the power in decision-making of under-served groups is intended to increase over the three stages, and the power in decision-making of university teams is intended to decrease over the three stages.

## Reflections prior to the project

This box includes challenges for community organisation teams and challenges for university teams, which led to the need identified.

Challenges for community organisation teams:

- Limited understanding of research opportunities
- Limited time
- Limited resources
- Competing priorities

Challenges for university teams:

- Limited understanding of under-served groups’ needs / priorities
- Difficulty engaging effectively with community organisations

Need identified:

- Time and resources to develop partnerships between community organisation teams and university teams

## Public involvement project underpinned by a knowledge mobilisation approach

This box summaries members of the project team, the co-produced activities and outputs, and the academic-led output. Arrows are used to indicate that the co-produced activities and outputs underpinned the academic-led output.

Project team:

- Community organisation teams involving public contributors from:
  - Expert Citizens
  - Voluntary Action Stoke on Trent
  - Leeds Involving People
  - Healthwatch Leeds
- University teams involving Patient and Public Involvement and Engagement staff and academics from:
  - Keele University
  - University of Leeds

Co-produced activities and outputs:

- Community conversations: community conversations with each of the four community organisations.
- Community-focused dissemination: creative and accessible outputs (e.g. artwork) and two dissemination events.
- Lessons learned: learning for future engagement with community organisations and under-served groups.

Academic-led output:

- Academic-focused dissemination: paper reporting the approach to partnership building, the community conversation findings, and lessons learned.

## Future projects

This box indicates that future research projects are intended to include participatory projects co-produced with under-served groups.

# Image description of Figure 3: Expert Citizens community conversation visual notes

Visual notes from the Expert Citizens community conversation by B. Mure, which consist of text and cartoons in green, purple, and black.

The title states ‘Welcome to our community conversation’.

There are four subtitles, each positioned near text as follows.

## Partnerships

- Importance of just being with people.
- Change doesn’t happen overnight, but we can take small steps together.
- Manage expectations – if change can’t happen need to explain why.
- Opinions have to feel valued, otherwise it feels pointless.
- Who is asking the questions?
- If someone is in a shirt and tie, you can feel like an interview.
- It affects your answers, you feel like you have to say ‘the right thing’.
- Location is important – walking through an open office is uncomfortable.
- We’re not exhibits.
- Build partnerships in an informal way: drop-in sessions for a chat and a brew.
- We are all survivors of systemic oppression.

## Finding out about health

- Two camps of thinking.
- I want to hear from a health professional.
- I use Dr Google or ask family and friends.
- Can’t always get appointment: ordered prescriptions online but it’s really expensive.
- Hard to get mental health information help that matters.
- What care and information is there for people on waiting lists?

## Accessibility

- We’re not tick boxes.
- People can’t keep using our experiences (for free!) and disappearing without anything ever happening!
- Sometimes notes are not up-to-date or accurate and we can’t access them.
- If you have to sit in that space multiple times you won’t give any more.
- Treat the whole person – don’t just manage the symptoms.
- Often I know more than the doctor.
- Searching online…
- I look for the NHS logo.
- I don’t always trust institutions…
- Digital divide – need to have information online and paper, maybe in video form or audio, Braille or translated.
- Use humour and creativity.

## Involving people in research

- Who do you trust with health information?
- People with lived experience or expertise.
- I don’t even trust my support nurse.
- What do you want to know about clinical research?
- I want to see the change the research is there to provide: it’s motivating!
- Is it sitting on a dusty shelf somewhere?
- It’s good to be valued by organisations.
- Two way value – researchers learn from us!
- Time and experience is valuable and should be compensated.
- Most people prefer face-to-face conversation to surveys and forms.
- We need to know outcomes and be given chance for feedback!

# Image description of Figure 4: Voluntary Action Stoke on Trent community conversation visual notes

Visual notes from the Voluntary Action Stoke on Trent community conversation by B. Mure, which consist of text and cartoons in blue, purple, and black.

The title states ‘Community health chat at Temple Methodist Church, Fenton and is next to the following text.

- People come here because it’s free and the church doesn’t judge.
- It’s nice to come and just connect!

There are four subtitles, each positioned near text as follows.

## Partnerships

- Would you speak to your GP? How easy is it?
- Is that one a joke?
- Depends on who you have and if you get in.
- Sometimes you have to make an appointment to get an appointment.
- You are number 12 in the queue…
- My prescription used to be 2 months supply… Then down to one.
- By the time I’ve got it I have to order it again.
- My family really help me.
- Can I just ask… we’re not sure who you are and what you’re doing?

## Finding out about health

- It just doesn’t work.
- I was a carer for 50 years so have background knowledge… I would really struggle without it.
- We’re so lucky in this country to have access to so much health info through the NHS… It’s not as easy in other countries.

## Accessibility and language

- Dyslexia friendly information.
- I need more time to discuss concerns and options.
- Living with HIV in the 80s and 90s, it was vital to find out information from reliable sources.
- How much do you agree?
- One of the biggest barriers to accessing health research is language that is too complex.
- Maybe for some – and it might encourage wider research participation.
- We need free Wi-Fi – could it be done through TV licence?
- Support and training.

## Involving people in research

- Taking research to people you want to reach would help.
- I would not know where to start looking for this stuff and I would love to take part.
- The animals at the top know what’s going on – the ones at the bottom have no idea. More information needs to be filtered down.

# Image description of Figure 5: Leeds Involving People community conversation artwork

Colourful artwork by Thomas Tickner representing the Leeds Involving People community conversation. The artwork includes cartoon people and rivers coming down from mountains and leading out to the sea.

The artwork includes the following text, which highlights the views of the community conversation attendees:

- We are community.
- We need access.
- We’re not represented.
- We need to be heard.
- We need a new path.
- An outdated system bypasses us all.
- Remember we’re diverse. So are our needs.

The artwork also includes a speech bubble coming out of a healthcare facility that states “We’re listening.”

Overall, the illustration aims to capture the community conversation attendees’ willingness to contribute to health research and recognition of the benefits of doing so, while also highlighting that a new path, which is flexible and tailored to diverse people’s needs, is needed to enable them to contribute to health research.

# Image description of Figure 6: Healthwatch Leeds community conversations combined posters

Four green and pink posters by Buttercrumble representing the Healthwatch Leeds community conversations. All the posters include cartoons, icons, text, and the Healthwatch Leeds, University of Leeds, NIHR Leeds Biomedical Research Centre and Leeds Teaching Hospital NHS Trust logos. Each poster is summarised below.

## How to get individuals and communities involved

- Improve access.
- Follow-up for feedback.
- Give thanks.
- Offer flexible participation options.
- Reach out via GPs and community groups.
- Explain the benefits.

## How do we get involved with research and who do we trust?

Chinese

- Increased knowledge.
- We use Chinese websites for health information.
- I can make a difference.

Bangladeshi

- I trust my GP for reliable health info.
- I want to make a positive impact.

Care at home

- I trust the NHS.
- Research is relevant.

Visually impaired

- We want to give back to the community.
- I want to learn more about my condition.

## Barriers and how to remove them

Care at home

- Barriers include the digital divide and lack of access.
- Strategies for addressing the barriers include reaching into communities and face-to-face communication.

Visually impaired

- Barriers include accessibility problems (e.g., sending printed letters)
- Strategies for addressing the barriers include in-person activities and one-to-one to support.

## Barriers and how to remove them

Chinese

- Barriers include language barriers and inaccuracies in translated information.
- Strategies for addressing the barriers include using interpreting services, focus groups, and increasing knowledge.

Bangladeshi

- Barriers include language and cultural barriers and not being aware of research opportunities.
- Strategies for addressing the barriers include working together with Healthwatch Leeds and carers/family members.
