# Supplementary material for: Building trust and inclusion with under-served groups: a public involvement project employing a knowledge mobilisation approach
Source: Res Involv Engagem. 2024 Nov 11;10:122. doi: 10.1186/s40900-024-00647-2 (PMC11555807; doi:10.1186/s40900-024-00647-2)
Supplement: Supplementary file 3 — Additional file 3 [file 40900_2024_647_MOESM3_ESM.docx]

Building trust and inclusion with under-served groups: a public involvement project employing a knowledge mobilisation approach

Additional file 3: Community conversation resources

# Expert Citizens co-produced framework

| **Finding out about health** | **WHAT** information needs to be shared? | **How** do you feel about how health information is shared with you? | **Where** do you go to find out health information?  Other thoughts? |
| --- | --- | --- | --- |
| **Accessibility/**  **language** | **WHO** do you trust to share knowledge/info about health research and why? | **What** are the best ways to share information about health/research?  How should we share the findings from our work/this community conversation and who with? | **What** barriers stop you from being able to access information?  How should information be presented to make it more understandable? |
| **Involving people in research** | **How** do you feel about getting involved with health research?  e.g. Doing a survey about your health condition or taking part in an interview | **What** would you like to know about health research? | If we did a project about a health condition, **how** would you like to hear about it? |
| **Partnerships** | **HOW** should information be co-created?  **Who** do we need to share this with? | **How** can we work together?  **What** are the best ways for us to get to know each other and build partnerships? | **How** would you like to share information, problems, questions and ideas? |

# Voluntary Action Stoke on Trent (VAST) co-produced statements

## Finding out about health

1. Health information needs to help me to manage my own health condition.
2. Health information from the NHS is trusted information that I would read.
3. It is important for people to be able to access their individual health record.
4. I feel that I have to find out information myself to support my health needs/conditions.

## Accessibility and language

1. I trust those close to me (family, peers) and support organisations to provide me with information about health research more than the NHS.
2. Information on health research needs to be brief, easily understandable and illustrated with graphics.
3. One of the biggest barriers to accessing health research information is that the language used is too complex.
4. I need better access to the internet to help me access information about health research.
5. I want to have the option to receive information about health research in a way that suits me (letter, digital, meetings).
6. After this event, I want to hear about what the information you gathered will be used for.

## Involving people in research

1. It is important to be clear about the reasons why the research is being carried out and what impact it will have on the NHS and my community.
2. I want to know how my contribution has helped and that my views and experiences have been respected and included.
3. I need to be supported to take part in research – for example I need enough time to fill out questionnaires, I need to know that what I share is kept private and that I am safe, I want to know where meetings are taking place and how long they will last, and I need to know if I am going to be paid.
4. I don’t know where or how I can get involved in research or what type of research I can be a part of.
5. I don’t like surveys; I prefer meeting in person and developing trusted and respectful relationships with researchers over time.

## Partnerships

1. I would be happy to discuss health topics if the conversations were held in my local church/community centre/cafe​.
2. I would like to find out about health information from friends/family, social media (Facebook group, twitter), website (NHS, other), local groups (which ones – church, activity groups, choir etc), blogs, GP, poster, university, none of the above, radio.
3. I would like to get involved in discussions about health research but don't know how​.
4. I don't trust health researchers because....

# 3. Leeds Involving People co-produced slides summary

## Slide 1

Text in central thought bubble: ‘Question 1: **What do you think about health research?**’

Text in surrounding thought bubbles:

- ‘Is it something you want to be part of?’
- ‘Have you seen chances to join in?’
- ‘How do you feel about health research?’
- ‘What do you think health research is meant to do?’
- ‘Have you taken part before?’

## Slide 2

Text in central thought bubble: ‘Question 2: **We need to change the ways we work. What do you think needs to change, and how?**’

Text in surrounding thought bubbles:

- ‘Have you ever worked with other groups, like the local council, health services, or community groups?’
- ‘What things went really well?’
- ‘What things didn't go so well?’
- ‘What things should we be careful not to do?’
- ‘How can we work together with groups like this in a way that helps everyone and lasts a long time?’

## Slide 3

Text in central thought bubble: ‘**Who are we and What is Health Research?**’

Text in surrounding thought bubbles:

- ‘We work at the NIHR Leeds Biomedical Research Centre in Chapel Allerton’
- ‘Our researchers work in the community to raise awareness on different health conditions.’
- ‘Our research involves: New medicines, New technologies (Apps and surgical), New therapies (Physiotherapy)’
- ‘We have research sites across Leeds Teaching Hospitals Trust and the University of Leeds.’
- ‘We are managed through the University of Leeds, University of York and Leeds Teaching Hospitals Trust.’
- ‘We work with health and care services to improve the health of the nation through research.’

# Healthwatch Leeds session plan and note taking template summary

## Understanding the group’s experiences and needs

1. Where do you go to find out health information?

Prompt: find out community’s perception of a reliable source for health info. What stops them from being able to access health information?

1. What experience if any have you had in health research?

Prompt: Their own experience, what went well, as well as what didn’t work well for you.

## Removing barriers to getting involved in health research

1. How would you prefer to receive health research information?

For example, email, letter, phone call, word of mouth an accessible format: such as audio, large print, letter in your own language.

1. What ways would you like to get involved in health research and why does this way work for you?

For example, completing a survey, taking part in an interview or focus group.

1. What will help you to get involved with health research and how would you like to receive research findings?

Motivation: increase knowledge, share personal experience, understand treatments, side effects, alternatives.

Logistics: such as taxi, disabled access, translation etc.

Financial: expenses, recognition incentive.

Other: Ethics.

Research findings: i.e. report, infographic, easy read, video, event etc.

## Working together on health research

1. What areas of health and care would interest you/community?
2. Who do you trust to share information about health research and why?

For example, community group, GP, Healthwatch

1. What would be the best way for National Institute for Health Research to work with you/community?
