# Supplementary material for: Building trust and inclusion with under-served groups: a public involvement project employing a knowledge mobilisation approach
Source: Res Involv Engagem. 2024 Nov 11;10:122. doi: 10.1186/s40900-024-00647-2 (PMC11555807; doi:10.1186/s40900-024-00647-2)
Supplement: Supplementary file 4 — Additional file 4 [file 40900_2024_647_MOESM4_ESM.pdf]

LEEDS involving people

NIHR | Leeds Biomedical Research Centre

Community Conversations

NHS The Leeds Teaching Hospitals NHS Trust

UNIVERSITY OF LEEDS

"To me, research should represent the advancement and betterment of good health for all."

We want a place to discuss issues in accessing healthcare:
- Transport
- Appointment times
- Health issues

More people with lived experiences should be leading research

Allow a two-way flow of information

We need to be treated with respect and understanding

Researchers shouldn't:
- Assume
- Judge
- Dictate
- Be profit driven

Listen to us!

We don't want formal reports! We want:
- Pictures and videos
- Podcasts
- Someone to talk to

There should be more accessibility awareness

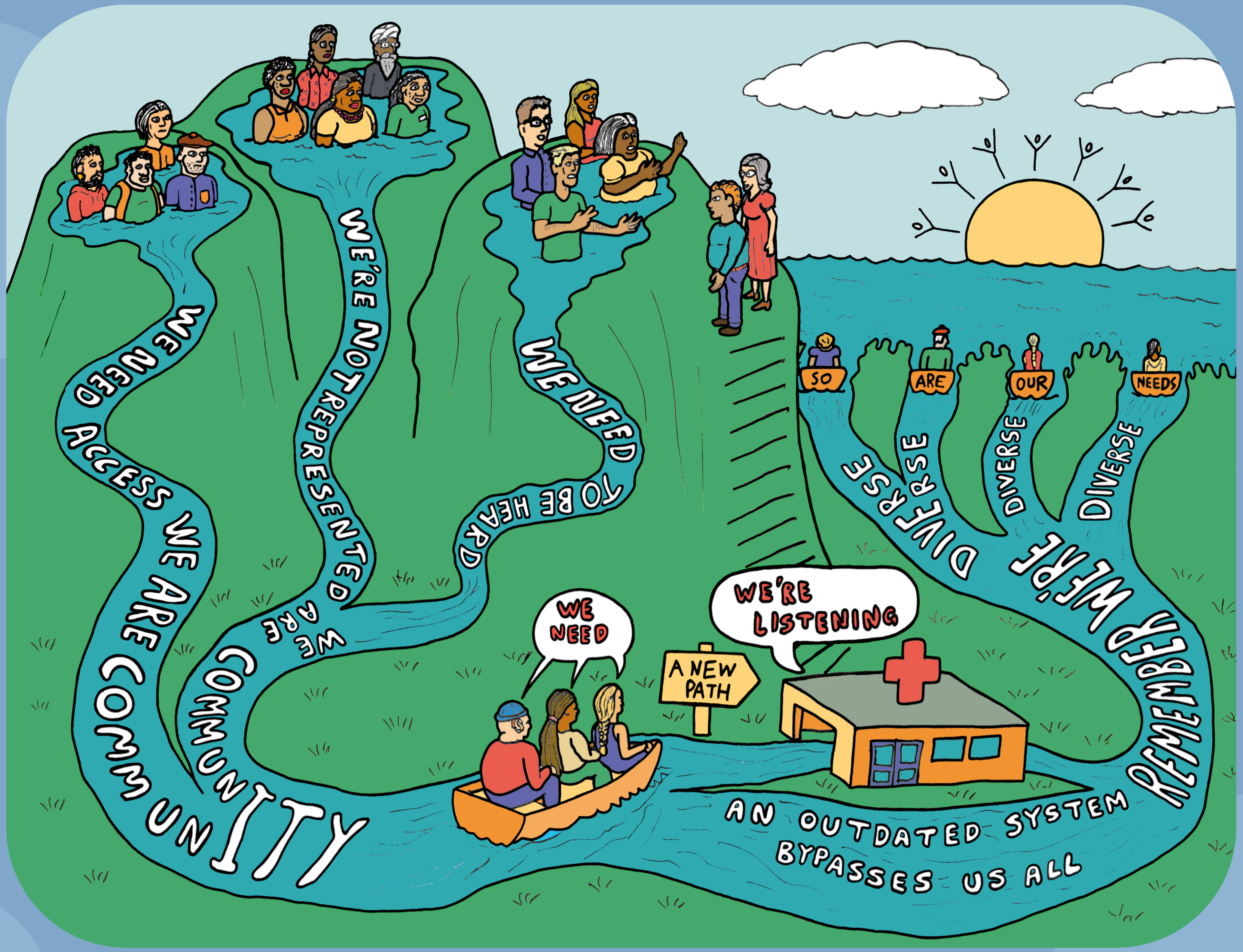

We want to keep working with you!

Contact us if you have any questions, or want to learn more:

Email: PPIBRC@leeds.ac.uk

Phone: 0113 392 4474
