# Supplementary material for: Building trust and inclusion with under-served groups: a public involvement project employing a knowledge mobilisation approach
Source: Res Involv Engagem. 2024 Nov 11;10:122. doi: 10.1186/s40900-024-00647-2 (PMC11555807; doi:10.1186/s40900-024-00647-2)
Supplement: Supplementary file 5 — Additional file 5 [file 40900_2024_647_MOESM5_ESM.pdf]

What are Community Conversations?

Health research isn't always easy for everyone to get involved in. There are different reasons for this, and the Community Conversations project was designed to explore these reasons further. Health research that is accessible for all is referred to as 'inclusive'.

To do this we worked with local people to create local solutions.

For our first step, we worked with community partners Leeds Involving People and Healthwatch Leeds to hold group meetings in community spaces. We called these 'Community Conversations'.

During the meetings, we listened to people's views of health research. This included identifying barriers to inclusion and ideas for making research more inclusive.

Get involved!

Want to get involved in making health research more inclusive?

Want to learn more about health research in Leeds?

You can contact any of the teams below.

Leeds BRC

- PPIBRC@leeds.ac.uk
- 0113 392 4474

Leeds Involving People

- info@leedsinvolvingpeople.org.uk
- 0113 237 4508

Healthwatch Leeds

- info@healthwatchleeds.co.uk
- 0113 898 0035

UNIVERSITY OF LEEDS

NHS The Leeds Teaching Hospitals NHS Trust

This project is funded by the National Institute for Health and Care Research (NIHR) [Programme Development Grant (NIHR205217) and supported by the NIHR Leeds Biomedical Research Centre. The views expressed are those of the author(s) and not necessarily those of the NIHR or the Department of Health and Social Care.

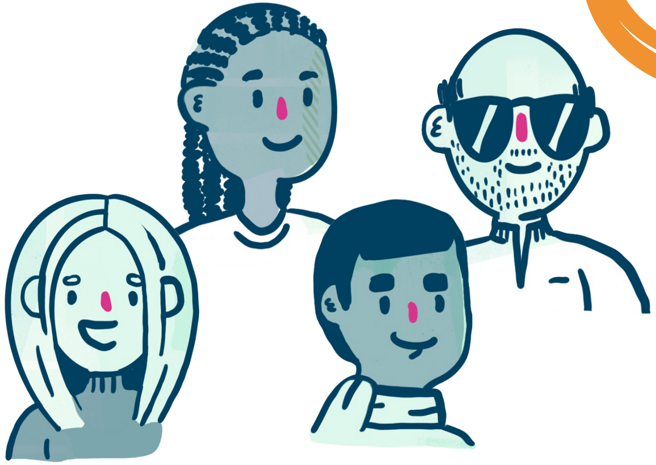

Community Conversations

Working together to make health research more inclusive

NIHR | Leeds Biomedical Research Centre

We held a big group Community Conversation with Leeds Involving People. Twenty-two people came to share their views with our team.

Key barriers identified

- Research does not focus on what matters most to local communities.
- It can be hard to find out about health research.
- Research can be scary and intimidating.

Key ideas to make research more inclusive

- Enable people with lived experience to plan and lead research.
- Go to community spaces to answer questions about research.
- Provide information in different languages and accessible formats.
- Pay or compensate everyone involved fairly for their time.
- Provide clear, timely and accessible feedback.

What are our next steps?

We want to carry on working with local people to help make health research more inclusive. Here are some of our ideas to continue the conversation:

- Work together to create effective accessibility training for researchers.
- Change how we recognise your involvement in health research - how do you want to be 'paid'?
- Bring our projects into community spaces. e.g. Ask the Researcher: an event where one of our researchers comes to a community space to share and answer questions about their research.

What do you think of our ideas? We welcome any and all feedback, and any ideas you might have yourself!

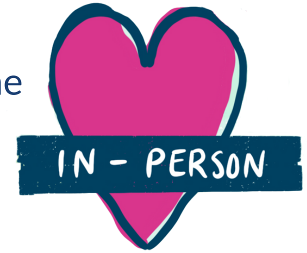

Contact our team: PPIBRC@leeds.ac.uk
or see the back of this flyer for more contact details.

We held four Community Conversations with Healthwatch Leeds. This allowed us to tailor the conversations to each of the groups, where a total of twenty-seven people joined the conversation.

The groups raised lots of similar points. For example, they all welcomed the idea of being involved in health research. They also raised points specific to their group.

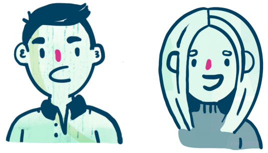

Ideas to make research more inclusive for specific groups

- Chinese: provide written feedback in Chinese.
- Bangladeshi: share research opportunities with them via their younger relatives.
- Care at Home: offer online research sessions with buddies.
- Visually Impaired: provide information in audio formats.
